# The mosquitoes of Armenia: review of knowledge and results of a field survey with first report of *Aedes albopictus*

**DOI:** 10.1051/parasite/2020039

**Published:** 2020-06-08

**Authors:** Lusine Paronyan, Lilit Babayan, Arsen Manucharyan, Dezdemonia Manukyan, Haykuhi Vardanyan, Gayane Melik-Andrasyan, Francis Schaffner, Vincent Robert

**Affiliations:** 1 National Center of Disease Control and Prevention, Ministry of Health 25 Heratsi str. Yerevan 0025 Republic of Armenia; 2 Francis Schaffner Consultancy Lörracherstrasse 50 4125 Riehen Switzerland; 3 National Centre for Vector Entomology, Institute of Parasitology, Vetsuisse Faculty, University of Zurich Winterthurerstrasse 266a 8057 Zürich Switzerland; 4 MIVEGEC Unit, IRD, CNRS, University of Montpellier 911, avenue Agropolis BP 64501 34394 Montpellier cedex 5 France

**Keywords:** Biodiversity, Culicidae, Invasive species, Vector, Malaria, Arbovirus

## Abstract

*Background*: In 2016, a field study was implemented in all Armenian provinces in order to update knowledge on the presence and distribution of both native and invasive mosquito species. Larvae and adult mosquitoes were sampled and identified on the basis of their morphology. Supplementary field surveys were performed in 2017–2018. *Results*: Between June 20 and July 12, 2016, 117 localities were visited. A total number of 197 sampling units were checked, of which 143 (73%) were positive for mosquitoes (with 1–6 species per sampling unit). A total number of 4157 mosquito specimens were identified to species or species complex level. Ten species represent first records for Armenia: *Aedes albopictus, Ae. annulipes, Ae. cataphylla, Ae. cinereus/geminus* (probably *Ae. cinereus*)*, Ae. flavescens*, *Anopheles plumbeus*, *Coquillettidia richiardii*, *Culex martinii*, *Cx. torrentium* and *Culiseta subochrea*. The invasive species *Ae. albopictus* was recorded in a single locality (Bagratashen) at the border point with Georgia, along the main road Tbilisi-Yerevan. This species was further recorded in 2017 and 2018, demonstrating its establishment and spread in north Armenia. These surveys confirm the presence of vectors of malaria parasites (in particular *An. sacharovi*) and West Nile virus (*Cx. pipiens*). *Conclusion*: The knowledge of the Armenian mosquito fauna is extended to a list of 28 species. The record of *Aedes albopictus,* an important potential vector of many arboviruses, has important implications for public health.

## Abstract (fr)

*Contexte* : En 2016, nous avons réalisé une étude sur le terrain dans toutes les provinces du pays dans le but d’actualiser la présence et la distribution des espèces de moustiques aussi bien natives qu’invasives. Les moustiques récoltés aux stades larvaires et adultes ont été identifiés sur des critères morphologiques. Des suivis additionnels ont été réalisés en 2017 et 2018. *Résultats* : Entre le 20 juin et le 12 juillet 2016, 117 localités ont été visitées. Au total, 197 unités de collecte ont été prospectées dont 143 (73%) se sont révélées positives pour les moustiques (avec de 1 à 6 espèces par unité de collecte). Au total, 4157 spécimens ont été identifiés au niveau de l’espèce ou du complexe d’espèces. Dix espèces ont été observées pour la première fois en Arménie : *Aedes albopictus, Ae. annulipes, Ae. cataphylla, Ae. cinereus/geminus* (probablement *Ae. cinereus*)*, Ae. flavescens*, *Anopheles plumbeus*, *Coquillettidia richiardii*, *Culex martinii, Cx. torrentium* et *Culiseta subochrea*. L’espèce invasive *Ae. albopictus* a été observée dans une seule localité (Bagratashen) à la frontière avec la Géorgie, sur l’axe routier principal Tbilisi-Yerevan. Cette espèce a également été observée en 2017 et en 2018, faisant la preuve de son installation et de sa diffusion dans le nord de l’Arménie. Ces suivis confirment la présence des vecteurs de *Plasmodium* humains (en particulier *An. sacharovi*) et du virus West Nile (*Cx. pipiens*). *Conclusion* : La connaissance de la faune culicidienne d’Arménie progresse, avec une liste actualisée à 28 espèces. L’observation d’*Aedes albopictus,* un important vecteur potentiel de nombreux arbovirus, a d’importantes implications en termes de santé publique.

## Introduction

The Republic of Armenia is a landlocked country in the Caucasus region of Eurasia. The territory is mostly mountainous with fast flowing rivers and few forests. The climate is highland continental, i.e. hot summers and cold winters. The country is characterised by a large variety of natural environments. Several distinct landscape zones are described: deserts, semi-deserts, dry steppes, steppes, woodlands, sub-alpine, and alpine lands. The conditions of these various natural landscape zones from the lowland plains to high mountains rely primarily on altitude, and shape the diversity of both flora and fauna, including insects. The country is divided into 11 provinces, of which 10 are regions (marzes) – Aragatsotn, Ararat, Armavir, Gegharkunik, Kotayk, Lori, Shirak, Syunik, Tavush, and Vayots Dzor – and the last one is Yerevan, the capital city.

The only mosquito-borne disease historically registered in Armenia is malaria. This disease was known to be highly endemic in the country from ancient times. It had persisted in Armenia throughout the centuries but was absent for 31 years, during the period of 1963–1993 [6, 24, 25, 28]. From 1994 to 2005, 4013 (1722 autochthonous) *Plasmodium vivax* malaria cases were registered [4, 7, 13] with the highest number in 1998 (1156) of which 542 were autochthonous; 31.3 cases per 100,000 inhabitants. No autochthonous cases have been reported since 2005 and the country was certified malaria-free by the WHO in 2011 [8].

Mosquito-borne arboviruses circulate in Armenia, without significantly threatening public and animal health. A large entomological survey conducted from 2003 to 2006 by the Armenian Institute of Epidemiology, Virology, and Medical Parasitology, identified 125 distinct strains of arboviruses isolated from 64,567 field-caught mosquitoes, including West Nile virus and others viruses like the Batai, Sindbis, Tahyna and Gheta viruses, without registered human or animal cases [19, 20].

In response to the *P. vivax* malaria outbreak, a national network of entomologists was established during the 1990s, and continues to operate today. Medical entomologists are active in all regions and Yerevan territorial branches of the National Center of Disease Control and Prevention (NCDC), with leadership at the National level – Reference Laboratory Center of NCDC at the Ministry of Health. Most entomological surveillance in Armenia focuses on anopheline mosquitoes, with registration of all stagnant water bodies throughout Armenia by routine investigation of stagnant water bodies for larvae and barns for adults every 10 days. Sampling techniques include dipping for larvae and adult resting catches with tubes from the walls of barns. Routine vector control activities rely mainly on the use of mosquito-larvivorous fish *Gambusia affinis*, and more rarely on insecticide spraying and the reduction of mosquito larval breeding sites [7].

The anopheline species, locally named “malaria mosquitoes”, are the most studied mosquitoes in Armenia. The *Anopheles maculipennis* complex is represented locally by two species. The first, *An. sacharovi* Favre, was the main malaria vector, with a marked anthropophilic biting behaviour. It is present in regions where rice is cultivated, in the central part of the Ararat Valley. The second, *An. maculipennis s.s.* Meigen, is widely distributed in the whole country. It is more zoophilic than *An. sacharovi* and therefore considered only a secondary malaria vector. In the late 1950s and the early 1960s, the *An. maculipennis* complex was thought to be eliminated from most of Armenia, but the use of insecticide was discontinued in the late 1960s and the numbers of *An. maculipennis s.s.* were restored, whereas *An. sacharovi* was not detected before the late 1990s. Several surveys of this complex were performed in the 1990s and 2000s using molecular methods for species differentiation [17, 23, 31].

VectorNet is a joint project of the European Centre for Disease Prevention and Control (ECDC) and the European Food Safety Authority (EFSA) supporting the collection of distribution data on vectors of pathogens, related to both human and animal health. An international VectorNet field mission reported the presence of *Aedes aegypti* (Linnaeus) in Georgia at 20 km north of the Georgia-Armenia border in September 2015 [1]. Here, we report results of another VectorNet field mission conducted in 2016 in Armenia with the aim of improving knowledge of the under-studied mosquito fauna (i.e. culicines) and assessing the presence and distribution of invasive mosquitoes, with special focus on the main arbovirus vectors present in the south Caucasus and Middle East, *Ae. aegypti, Ae. albopictus* (Skuse), *Culex perexiguus* Theobald and *Cx. tritaeniorhynchus* Giles [12]. This field study was conducted as a snapshot survey (inspection of a maximum of sites within a short period of time in June–July 2016). To confirm and complete the observations, the survey was prolonged in August–October 2016. Finally, we report results of longitudinal surveys targeting West Nile vector species performed in 2017 and 2018 as part of the ECDC project “Development of a Tool to Appraise and Compare Vector Control Strategies against West Nile Fever in Europe” with the specific aim of evaluating mosquito population dynamics and relative abundance.

## Materials and methods

**Figure 1:**
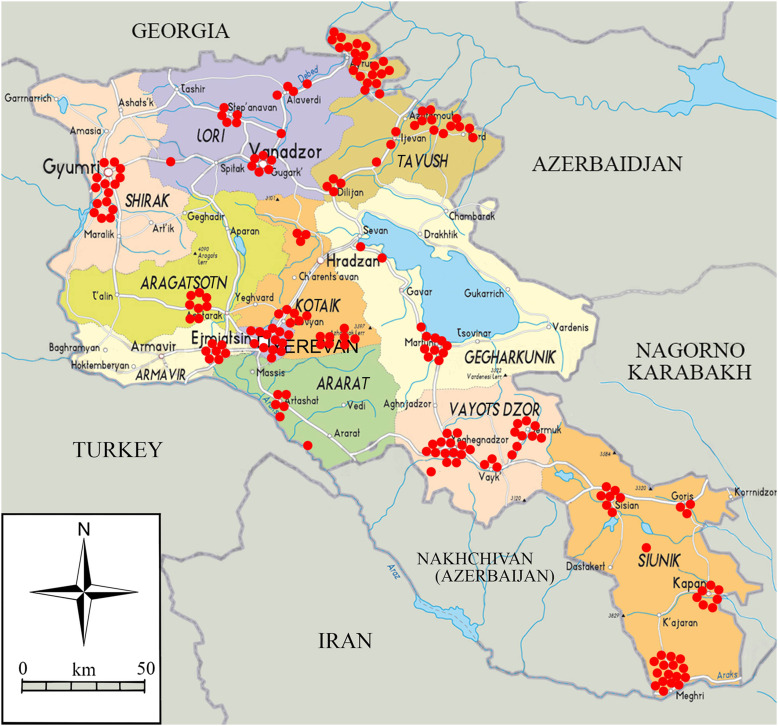
Location of the sampling sites in Armenia, June 20 to July 10, 2016.

In 2016, a snapshot field sampling campaign was performed from June 20 to July 12, 2016, in all 11 administrative units of Armenia, namely the 10 regions (Aragatsotn, Ararat, Armavir, Gegharkunik, Kotayk, Lori, Shirak, Syunik, Tavush, Vayotz Dzor) and the capital city of Yerevan (Fig. 1). A longitudinal survey focusing on a selected number of sites was performed in August–October 2016 as well as in March–November 2017 and April–October 2018, in almost all Armenian regions (except Shirak and Gegharkunik) and in Yerevan.

Classical entomological methods were used, based on protocols defined for VectorNet field studies [11]. We mainly collected immature aquatic stages by dipping/netting. Occasionally, adults were collected with baited traps: All-Weather LED EVS traps (BioQuip, USA) baited with CO_2_ as dry ice, CDC Light Traps (CDCLT: Model 512, John W. Hock Company, USA), and BG Sentinel II™ traps (BGS: Biogents, Germany) baited with BG-Lure™ (Biogents, Germany) and CO_2_ as dry ice. Also, simple ovitraps were used to collect eggs from container breeding *Aedes* species [11]. In addition, resting adult mosquitoes were caught in animal shelters using aspirators and tubes. CDCLT, EVS and BGS traps were operated during the night, from at least 2 h before sunset up to 2 h after sunrise. They were placed in safe areas, avoiding the traps being damaged by animals or stolen. EVS and CDCLT were hung with the trap entrance at 1.5 m high and BGS placed on the ground. Finally, human landing catches was performed at daytime in Bagratashen (targeting *Ae. albopictus*). The collections were performed at varying frequency detailed in Tables S1–S4. Field data were reported using the VECMAP^®^ system (Avia-GIS, Belgium), in particular its mobile telephone application.

Entomological observations were analysed per sampling unit, defined as a collection of mosquitoes using the same collecting method at the same site. For instance, larval sampling was performed at several points of a large marsh within the same sampling unit. A sampling unit could be positive or negative for mosquitoes. Thus, sampling units can be multiple at one site, using for instance both resting adult collection and ovitrapping. Some localities also had several sites (village, city, etc.).

The larvae and adult specimens were identified based on standard morphological keys [5, 15, 26]. Molecular identification by DNA isolation and amplification of part of the mitochondrial cytochrome oxidase subunit I gene (mt COI) was performed for a single specimen, as described elsewhere [27]. Pupae were reared to obtain adults.

## Results

Our survey in 2016–2018 increased the number of mosquito species recorded in Armenia from 18 to 28. The presence of the invasive species *Ae. albopictus* was recorded at a single locality at a border point with Georgia, on the main road Tbilisi-Yerevan. *Anopheles sacharovi*, the main malaria vector in Armenia, was only found in the Ararat Valley.

### Historical records

Table 1 summarises published mosquito records (reference sources are given in the table). Up to 2013, 18 species were known to occur in Armenia. Five mosquito species of the Anophelinae subfamily were listed: *An. maculipennis s.l./s.s.*, *An. sacharovi*, *An. hyrcanus* (Pallas), *An. superpictus* Grassi and *An. claviger* (Meigen). As for the Culicinae subfamily, four genera were listed, namely *Aedes*, *Culex*, *Culiseta* and *Uranotaenia*. Four *Aedes* species were recorded: *Ae. caspius* Pallas which was found in many lowlands, *Ae. vexans* (Meigen) reported to be more widely distributed in various landscapes, *Ae. dorsalis* (Meigen) and *Ae. geniculatus* (Olivier) of which larvae were found in small natural reservoirs in the forest and forest-steppe zones where they were reported as aggressive biters. With six species, the genus *Culex* was highly represented, with *Cx. pipiens* Linnaeus and *Cx. theileri* Theobald as the most widely distributed species, *Cx. hortensis* Ficalbi and *Cx. modestus* Ficalbi only noted in the Ararat Valley, and *Cx. mimeticus* Noè and *Cx. territans* Walker only recorded in one study [18]. Two species of the genus *Culiseta* were listed, i.e. *Cs. longiareolata* (Macquart) found in the Ararat Valley only, and *Cs. annulata* (Schrank) more widely distributed. Finally, from the genus *Uranotaenia*, the species *Ur. unguiculata* Edward was reported from the Ararat Valley to occur in temporary pools and small ponds.

**Table 1:** Mosquito species/taxa reported to occur in Armenia in the literature, prior to 2016 (1 = presence record).

	Terteryan and Mirumyan [30]	Romi et al. [23]	Manukyan et al. [19]	Manukyan et al. [20]	WHO Europe [31]	Keshishyan et al. [17]	Manukyan et al. [18]
*Aedes* (*Och.*) *caspius*		1	1	1			1
*Aedes* (*Och.*) *dorsalis*							1
*Aedes* (*Dah.*) *geniculatus*	1						1
*Aedes* (*Adm.*) *vexans*	1						1
*Anopheles* (*Ano.*) *claviger*	1	1	1	1			
*Anopheles* (*Ano.*) *hyrcanus*			1	1			
*Anopheles* (*Ano.*) *maculipennis s.l*.	1	1	1	1	1	1	
*Anopheles* (*Ano.*) *maculipennis s.s*.		1			1	1	
*Anopheles* (*Ano.*) *sacharovi*		1			1	1	
*Anopheles* (*Cel.*) *superpictus*	1		1	1			
*Culex* (*Mai.*) *hortensis*				1			1
*Culex* (*Cux.*) *mimeticus*							1
*Culex* (*Bar.*) *modestus*							1
*Culex* (*Cux.*) *pipiens*	1	1	1	1			1
*Culex* (*Ncx.*) *territans*							1*
*Culex* (*Cux.*) *theileri*		1	1	1			1
*Culiseta* (*Cus.*) *annulata*	1						1
*Culiseta* (*All.*) *longiareolata*	1						1
*Uranotaenia* (*Pfc.*) *unguiculata*							1
Total	8	7	7	8	3	3	13

* Cited as *Culex* (*Ncx*.) *apicalis* Adams, 1903.

### Field mission June 20–July 10, 2016

**Figure 2:**
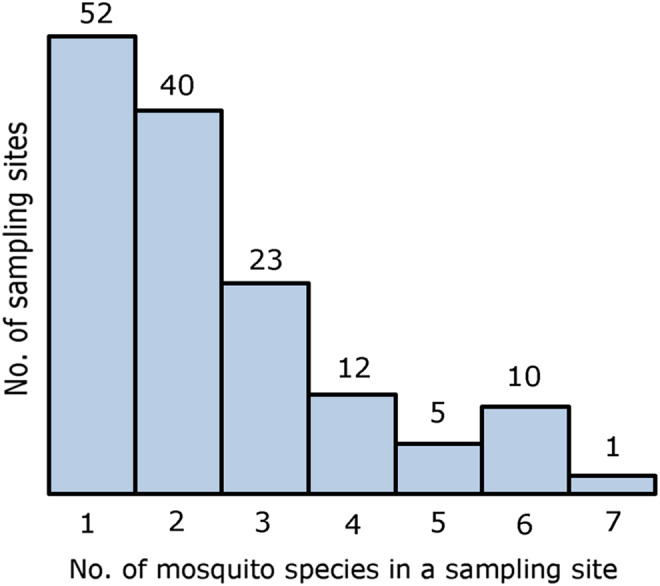
Number of sampling units that were found to be positive for mosquitoes (total = 143), according to the number of mosquito species observed in each sampling, June 20 to July 10, 2016. *Anopheles maculipennis s.l*. is considered a “species” when no precise species from the complex is listed.

A total number of 117 sites were visited (in all Armenian regions) and 197 sampling units were assessed, showing 143 to be positive for larvae and/or adults and 54 remaining negative (Tables 2 and 3, and Table S1). The positive sampling units harboured 1–6 mosquito species, with 37% (*n* = 53) showing monospecific mosquito fauna (Fig. 2). A total number of 4157 mosquito specimens were observed (3152 larvae and 1005 adults – caught or emerged from pupae), belonging to 24 species/taxa. Ten species are new records for Armenia: *Ae. albopictus*, *Ae. annulipes* (Meigen), *Ae*. *cataphylla* Dyar, *Ae*. *cinereus*/*geminus* Meigen/Peus, *Ae. flavescens* (Müller), *An*. *plumbeus* Stephens, *Coquillettidia richiardii* (Ficalbi), *Cx*. *martinii* Medschid, *Cx. torrentium* Martini and *Cs. subochrea* (Edwards).

**Table 2:** Number of positive sites per mosquito species recorded in Armenia in 2016, according to regions. Asterisk indicates a first record for Armenia.

	Aragatsotn	Ararat	Armavir	Gegharkunik	Kotayk	Lori	Shirak	Syunik	Tavush	Vayots Dzor	Yerevan	Total no. of sites	Total no. of specimens
*Aedes* (*Stg.*) albopictus*									1			1	2
*Aedes* (*Och*.) annulipes*				3		1				3		7	15
*Aedes* (*Och*.) *caspius*	3	4	3				5	2	1			19	211
*Aedes* (*Och.*) cataphylla*				2								2	2
*Aedes* (*Aed*.) cinereus/geminus*				1								1	7
*Aedes* (*Och.*) flavescens*		1		1			1					3	5
*Aedes* (*Dah.*) *geniculatus*					2				1	1		4	18
*Aedes* (*Adm.*) *vexans*	2	1	2			1		1	7	8		22	103
*Anopheles* (*Ano*.) *claviger*				2			2	3	4	3	2	16	85
*Anopheles* (*Ano.*) *maculipennis s.l*.	7	2	4		3	1	5	7	14	6	2	51	625
*Anopheles* (*Ano.*) *maculipennis s.s*.	4	1	2					1		1		9	31
*Anopheles* (*Ano.*) plumbeus*								1				1	10
*Anopheles* (*Ano.*) *sacharovi*			1									1	1
*Coquillettidia* (*Coq.*) richiardii*		2			1							3	7
*Culiseta* (*Cus.*) *annulata*	2		5				4	4	1		2	18	145
*Culiseta* (*All.*) *longiareolata*	1				1	1		8		3	2	16	396
*Culiseta* (*Cus*.) subochrea*	1		1	3	1		1	2	1			10	24
*Culex* (*Mai.*) *hortensis*	1			3	1	3	2	8	4	1		23	108
*Culex* (*Ncx.*) martinii*	1											1	1
*Culex* (*Cux.*) *pipiens*	5	1	6	4	4	8	5	15	15	3	5	71	1562
*Culex* (*Ncx.*) *territans*	1				1			1	1	1		5	41
*Culex* (*Cux.*) *theileri*	4	3	3	6	2		8	10	8	6	2	52	746
*Culex* (*Cux.*) torrentium*				1				1				2	4
*Uranotaenia* (*Pfc.*) *unguiculata*									2	1		3	8
Total	32	15	22	31	17	14	33	64	60	37	16	341	4157
Total no. of sites per region	10	6	6	12	17	17	15	34	40	27	13	197	

**Table 3:** Sampling effort of the surveys, according to period and sampling method.

	Larval sampling	Adult trapping	Human bait	Resting catch	Ovitrap	Total
Jun–Jul 2016	94	40	9	41	13	197
Aug–Oct 2016	19	9	1	0	0	29
Mar–Nov 2017	998	584	0	251	0	1833
Apr–Oct 2018	1302	26	2	753	0	2083
Total	2413	659	12	1045	13	4142

**Figure 3:**
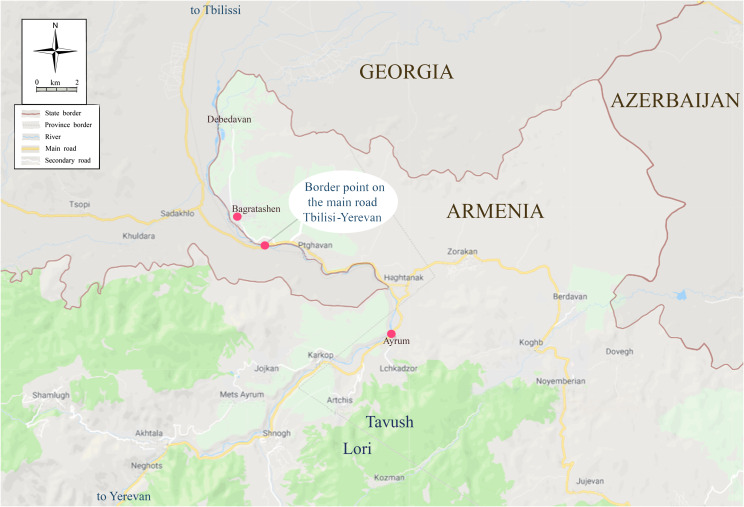
Location of the sites where *Aedes albopictus* was recorded in 2016–2018, north Armenia.

Two adult *Ae. albopictus* females were collected for the first time in Armenia on July 7, 2016, by netting around humans, 100 m from a bridge over the Debeb River (in Bagratashen, Lori Province, Fig. 3), which is the border point on the main Tbilisi-Yerevan road.

A unique and poorly preserved specimen of *Aedes* sp. (collected in Gegharkunik on June 24, 2016) morphologically identified as “cataphylla/leucomelas/punctor”, revealed a COI sequence (GenBank sequence submission number MT457072, Specimen 16-116ARM) showing 100 to 98.7% similarities with *Ae. leucomelas* (Meigen), 99.5–97.7% similarities with *Ae. cataphylla*, and 90.6% similarity with *Ae. punctor* (Kirby). Because *Ae. cataphylla* was recorded in Gegharkunik province (Table 2 and Table S1) and *Ae. leucomelas* was never recorded in Armenia, we assign this specimen to *Ae. cataphylla* until additional and better preserved specimens can be clearly assigned to *Ae. leucomelas*.

Among the *Anophelinae*, *An. maculipennis s.s.* and *An. claviger* were the most prevalent species and were distributed in most of the regions. *Anopheles sacharovi* was observed in the Ararat Valley, and *An. plumbeus* was observed as larval stages in tyres in Syunik Province.

Among the Culicinae, the most common and numerous *Aedes* species was *Ae. caspius*, found in many regions except the highlands of the Sevan basin and Syunik; *Ae. vexans* was observed in all climatic zones and in various landscape conditions, at all places with temporary and subpermanent reservoirs. Larvae of *Ae. geniculatus* were found in small reservoirs temporarily filled with water, and in the forest and forest-steppe zones where adults are aggressive biters. Concerning the genus *Culiseta*, *Cs. longiareolata* was found in the Ararat, Aragatsotn, Kotayk and Gegharkunik regions, and in the city of Yerevan, and *Cs. annulata* was distributed in all types of landscapes in Armenia. Within the genus *Culex,* the most widely distributed species were *Cx. hortensis*, *Cx. theileri* and *Cx. pipiens*; these three species were found in the lowland and foothill parts of the semi-desert area, as well as in forest and forest-steppe areas. *Culex martinii*, *Cx. territans* and *Cx. torrentium* presented lower abundances. *Uranotaenia unguiculata* was found in the Armavir, Kotayk, Ararat and Vayots-Dzor regions, in temporary pools or small ponds.

### Field mission August 3–October 30, 2016

A total number of 19 sites were visited (in all regions except Aragatsotn, Ararat, Armavir and Syunik), and 29 sampling units were evaluated with 24 proving to be positive for mosquitoes and 5 negative (Table S2). The positive sampling units harboured 1–4 mosquito species and a total number of 166 mosquitoes (119 larvae and 47 adults) from 9 species/taxa. *Aedes albopictus* was again observed at the border point on the main road Tbilisi-Yerevan close to Bagratashen village (Fig. 3), with 14 and 6 adults caught by human landing catch on August 14 and September 28, respectively (30 min to 1 h catch during the day time).

### Field mission March 6–December 12, 2017

A total number of 107 sites were visited (in all regions except Gegharkunik and Shirak), and 1393 sampling units were evaluated with 1379 proving to be positive for mosquitoes and 14 negative (Table S3). The positive sampling units harboured 1–8 mosquito species. A total number of 19,911 mosquitoes (16,305 larvae and 3606 adults) from 18 species were collected. *Aedes albopictus* was observed at the same location as in 2016 as biting adults (6, 5, and 7 females on June 12, August 15, and September 20, respectively, during the day time) (Fig. 3).

### Field mission April 3–October 31, 2018

In 2018, the same sites were visited as in 2017, and 2085 sampling units were evaluated with 1212 found to be positive and 873 negative (Table S4). Positive sampling units included 1 to 5 mosquito species. A total number of 12,116 mosquitoes (7290 larvae and 4826 adults) were collected. *Aedes albopictus* was confirmed established at Bagratashen village by adult catches (29 females from August 22 to 24 during the day time) and for the first time also in Ayrum village (Tavush Province, Fig. 3), 10 km away from Bagratashen along the road to Yerevan, as larval and adult stages (3 larvae and 2 adults observed on August 23).

## Discussion

Our results are important in terms of biodiversity but also in terms of public health. The most abundant mosquito species in our study in Armenia were found to be *Ae. caspius*, *Cx. pipiens* and *An. maculipennis*, consistent with previous studies [18–21, 23]. Ten mosquito species are new records for Armenia. This is a notable increase with regard to the total number of 28 species now reported (see full list in Appendix). The number of anopheline species has changed little from 5 to 6, with the addition of *An. plumbeus*; this contrasts with the 9 species of Culicinae and confirms that the Culicinae had been neglected in Armenia prior to our studies. Our results also demonstrate the value of a snapshot field study, when performed at a suitable period and over a wide range of environments.

Interestingly, the field mission in 2016 was organised with the knowledge of the presence of *Ae. aegypti* at the border point with Georgia, where it had been recorded in 2015, 20 km away in a direct line towards the north [1]. Interestingly, no *Ae. aegypti* were observed, but instead *Ae. albopictus*. This unexpected observation means that we should expand some results geographically only with extreme caution.

The observation of one female only for *Aedes cinereus/geminus* is unfortunate because only males allow for a reliable differentiation between these two sibling species. Considering the distribution of the two species, the most probable to occur in Armenia is *Ae. cinereus*, since this species is present in Turkey and Georgia, in contrast to *Ae. geminus* ([22] and Table S5).

Although our sampling effort was limited, the absence of records for some mosquito species is surprising. However, future inventories will probably add new species. Table S5 lists mosquito species recorded in all surrounding countries (Georgia, Azerbaijan, north-western Iran, and eastern Turkey/Anatolia), which leads us to pay special attention to certain vector species: *Ae. aegypti* (recorded in Georgia and Turkey/Anatolia), *Cx. perexiguus* (recorded in north-western Iran) and *Cx*. *tritaeniorhynchus* (recorded in Georgia, Azerbaijan and Turkey/Anatolia).

*Aedes dorsalis* was previously observed in Armenia [18], but not in our 2016–2018 surveys, which is puzzling. It is highly possible that, in the previous studies, *Ae. dorsalis* had been misidentified and confused with white morphotypes of *Ae. caspius*, as proposed by Günay et al. [14] for specimens from the Ararat Valley in Turkey [3].

*Aedes albopictus* is an important potential vector of many arboviruses. A recent introduction into Armenia with Georgian geographic origin is most probable but not demonstrated. We collected evidence of the establishment of this invasive species with consecutive records made at the border point along the Tbilisi-Yerevan road in 2016 (from August 11 to September 28), in 2017 (June 13), and in 2018 (August 23). The potential of dispersion within Armenia along this main road axis must not be underestimated. In fact, we recorded the species in 2018 in Ayrum village, 10 km away from the border point. Close follow-up of the current dispersion in Armenia will be much needed during the coming years. This requires an adjustment of entomological surveillance activities in Armenia. Medical entomologists are active only in the NCDC. Routine entomological surveillance is carried out all over the country with the following objectives: entomological monitoring, implementation of vector control activities with special measures at points of entry, prevention of endemic vector-borne infectious diseases, scientific and applied research on vectors, and communication to raise awareness of vector control measures among the population. Up to 2016, entomological surveillance in Armenia focused on anophelines only (“malaria mosquitoes” versus “non-malaria mosquitoes”). Methodological tools for entomological surveillance were updated, and new sampling protocols and reporting forms were adopted; this allows us now to also efficiently survey culicine mosquitoes. All these improvements show the added value for public health in terms of knowledge improvement and capacity building for an up-to-date mosquito surveillance system. This will help to prevent and mitigate mosquito-borne diseases in Armenia.

## Conclusion

Knowledge of the mosquito fauna of Armenia is progressing. It now encompasses 28 species, with 10 species recorded for the first time in the present study. The presence of *Ae. albopictus* in the extreme north of Armenia, along an important access road (Tbilisi-Yerevan) is a major finding and holds implications for public health in terms of the risk of arbovirus transmission. Following this survey, entomological surveillance was reorganised in Armenia to include invasive mosquitoes in addition to the anopheline malaria vectors.

## Supplementary material

Supplementary material is available at https://www.parasite-journal.org/10.1051/parasite/2020039/olm *Tables S1–S4*: Detailed mosquito collection data in Armenia for June to July 2016, August to October 2016, March to November 2017, and April to October 2018, respectively. (EXCEL file) *Table S5*: Mosquito species recorded in Armenia and in the surrounding countries. (PDF file)

## Appendix

Annotated checklist of the 28 mosquito species of Armenia

**Table:**

**Family Culicidae Meigen, 1818**
**Subfamily Anophelinae Grassi, 1900**
**Genus *Anopheles* Meigen, 1818**
**Subgenus *Anopheles* Meigen, 1818**
1 – *An.* (*Ano*.) *claviger* (Meigen, 1804) (see Note 1)
2 – *An.* (*Ano*.) *hyrcanus* (Pallas, 1771)
***Anopheles maculipennis* Meigen, 1818 Species Group** (see Note 2)
3 – *An*. (*Ano*.) *maculipennis s.s*. Meigen, 1818
4 – *An*. (*Ano*.) *sacharovi* Favre, 1903
5 – *An*. (*Ano*.) *plumbeus* Stephens, 1828
**Subgenus *Cellia* Theobald, 1902**
6 – *An*. (*Cel*.) *superpictus* Grassi, 1899 (see Note 3)
**Subfamily Culicinae Meigen, 1818**
**Tribe Aedini Neveu-Lemaire, 1902**
**Genus *Aedes* Meigen, 1818**
**Subgenus *Aedes* Meigen, 1818**
7 – *Ae*. (*Aed*.) *cinereus* Meigen, 1908 (see Note 4)
**Subgenus *Aedimorphus* Theobald, 1903**
8 – *Ae.* (*Adm.*) *vexans* (Meigen, 1830) [*Aedimorphus vexans* (Meigen)] (see Note 5)
**Subgenus *Dahliana* Reinert, Harbach & Kitching, 2006**
9 – *Ae.* (*Dah.*) *geniculatus* (Olivier, 1791) [*Dahliana geniculata* (Olivier)]
**Subgenus *Ochlerotatus* Lynch Arribálzaga, 1891**
10 – *Ae.* (*Och.*) *annulipes* (Meigen, 1830) [*Ochlerotatus annulipes* (Meigen) *s.l.*]
11 – *Ae.* (*Och.*) *caspius* (Pallas, 1771) *s.l*. [*Oc. caspius* (Pallas) *s.l*.] (see Note 6)
12 – *Ae*. (*Och*.) *cataphylla* Dyar, 1916 [*Oc. cataphylla* (Dyar)]
13 – *Ae.* (*Och.*) *flavescens* (Müller, 1764) [*Oc. flavescens* (Müller)]
14 – *Ae*. (*Och*.) *dorsalis* (Meigen, 1830) [*Oc. flavescens* (Meigen)] (see Note 7)
**Subgenus *Stegomyia* Theobald, 1901**
15 – *Ae.* (*Stg.*) *albopictus* (Skuse, 1895) [*Stegomyia albopicta* (Skuse)]
**Tribe Culicini Meigen, 1818**
**Genus *Culex* Linnaeus, 1758**
**Subgenus *Barraudius* Edwards, 1921**
16 – *Cx.* (*Bar.*) *modestus* Ficalbi, 1889
**Subgenus *Culex* Linnaeus, 1758**
17 – *Cx.* (*Cux.*) *mimeticus* Noè, 1899
18 – *Cx.* (*Cux.*) *pipiens* Linnaeus, 1758 (see Note 8)
19 – *Cx.* (*Cux.*) *theileri* Theobald, 1903
20 – *Cx.* (*Cux.*) *torrentium* Martini, 1925
**Subgenus *Maillotia* Theobald, 1907**
21 – *Cx.* (*Mai.*) *hortensis* Ficalbi, 1889
**Subgenus *Neoculex* Dyar, 1905**
22 – *Cx*. (*Ncx*.) *martinii* Medschid, 1930
23 – *Cx.* (*Ncx.*) *territans* Walker, 1856 (see Note 9)
**Tribe Culisetini Belkin, 1962**
**Genus *Culiseta* Felt, 1904**
**Subgenus *Allotheobaldia* Broelemann, 1919**
24 – *Cs.* (*All.*) *longiareolata* (Macquart, 1838)
**Subgenus *Culiseta* Felt, 1904**
25 – *Cs.* (*Cus.*) *annulata* (Schrank, 1776)
26 – *Cs.* (*Cus.*) *subochrea* (Edwards, 1921)
**Tribe Mansoniini Belkin, 1962**
**Genus *Coquillettidia* Dyar, 1905**
**Subgenus *Coquillettidia* Dyar, 1905**
27 – *Cq.* (*Coq.*) *richiardii* (Ficalbi, 1889)
**Tribe Uranotaeniini Lahille, 1904**
**Genus *Uranotaenia* Lynch Arribálzaga, 1891**
**Subgenus *Pseudoficalbia* Theobald, 1912**
28 – *Ur.* (*Pfc.*) *unguiculata* Edwards, 1913

Note 1 Member of the *An. claviger* complex. *Anopheles petragnani* is absent from the Caucasus.

Note 2 *An. maculipennis* complex. Two species only are present: *An. maculipennis s.s*. and *An. sacharovi*. Ribosomal RNA gene sequences are available for both species [10]. *Anopheles maculipennis s.s*. is the most abundant *Anopheles* species in Armenia, and *An. sacharovi* is an important malaria vector in the Middle East [29].

Note 3 *An. superpictus*. Old record [30] at larval stage. Identification likely reliable because this species belongs to the *Cellia* subgenus. Complex of several species observed in Iran, listed as species A and B by Harbach [16].

Note 4 *An. cinereus* is more likely in Armenia because it has been found in the region, unlike *Ae. geminus*.

Note 5 *Ae. vexans* subspecies. Presence of *Ae. vexans vexans.*

Note 6 *Ae. caspius* subspecies. Presence of *Ae. caspius caspius.* Abundant in lowland plains.

Note 7 *Ae. dorsalis*. Uncertain for presence; possible misidentification. Previous records in Armenia [18] at densities higher for *Ae. dorsalis* than for *Ae. caspius*, which is suspect. Recent records in the Ararat Valley (right bank, Turkey [2]) are now considered to be a “pale form” of *Ae. caspius* (Alten & Günay, Personal communication). Not observed in our studies.

Note 8 *Cx. pipiens* complex. Evidence of presence of the anthropophilic *Cx. pipiens* biotype molestus, the most abundant mosquito species in Armenia. Absence of *Cx. quinquefasciatus* (present in southern half of Iran [9]).

Note 9 *Cx. territans*. Recorded as *Cx. apicalis* Adams, 1903 (a north American species) in [18].
