# Supplementary material for: The mosquitoes of Armenia: review of knowledge and results of a field survey with first report of Aedes albopictus
Source: Parasite. 2020 Jun 8;27:42. doi: 10.1051/parasite/2020039 (PMC7278218; doi:10.1051/parasite/2020039)
Supplement: Supplementary file 2 — Table S5: Mosquito species recorded in Armenia and in the surrounding countries. (PDF file) [file parasite-27-42-s2.pdf]

Supplementary file. Paronyan et al (2020) The mosquitoes of Armenia: review of knowledge and results of a field survey with first report of *Aedes albopictus. Parasite.*

Table S5: Mosquito species recorded in Armenia and in the surrounding countries. A number between brackets indicates record and refers to the bibliographic list below the table.

	Armenia	Georgia	Azerbaijan (including Nakhitchevan)	Iran (only the 3 Provinces of north-western Iran: Ardebil, East Azerbaijan and West Azerbaijan)	Turkey (Anatolia only)
Ae. (Stg.) aegypti		[1]			[1]
Ae. (Stg.) albopictus	[herein]	[1]			[1]
Ae. (Och.) annulipes	[herein]				[1]
Ae. (Och.) caspius	[herein]	[1]	[1]	[2,3,4,5]	[1]
Ae. (Och.) cataphylla	[herein]		[1]		[1]
Ae. (Aed.) cinereus	[herein]	[1]			[1]
Ae. (Och.) communis					[1]
Ae. (Stg.) cretinus		[1]			[1]
Ae. (Och.) cyprius					[1]
Ae. (Och.) detritus					[1]
Ae. (Och.) dorsalis	[herein]				[1]
Ae. (Dah.) echinus					[1]
Ae. (Och.) excrucians		[1]			[1]
Ae. (Och.) flavescens	[herein]			[5]	[1]
Ae. (Dah.) geniculatus	[herein]	[1]	[1]	[2,4]	[1]
Ae. (Och.) intrudens		[1]			
Ae. (Rus.) lepidonotus					[1]
Ae. (Och.) leucomelas					[1]
Ae. (Acy.) phoeniciae					[1]
Ae. (Och.) nigrocanus					[1]
Ae. (Och.) pulcritarsis		[1]	[1]		[1]
Ae. (Och.) pullatus					[1]
Ae. (Och.) punctor		[1]			[1]
Ae. (Rus.) refiki					[1]
Ae. (Rus.) rusticus					[1]
Ae. (Och.) sticticus		[1]			
Ae. (Och.) surcoufi			[1]		
Ae. versicolor			[1]		
Ae. (Adm.) vexans	[herein]	[1]	[1]	[3,4,5]	[1]
Ae. (Acy.) zammitii					[1]
An. (Ano.) algeriensis		[1]			[1]
An. (Cel.) cinereus					[1]
An. (Ano.) claviger	[herein]	[1]	[1]	[2,3,4,5]	[1]

An. (Ano.) hyrcanus	[herein]	[1]	[1]	[3,4,5,6]	[1]
An. (Ano.) maculipennis s.s.	[herein]	[1]	[1]	[4,6]	[1]
An. (Ano.) melanoon		[1]	[1]		[1]
An. (Ano.) messeae					[1]
An. (Ano.) persiensis			[1]		
An. (Ano.) marteri					[1]
An. (Ano.) plumbeus	[herein]	[1]	[1]		[1]
An. (Ano.) pseudopictus				[3,4]	
An. (Cel.) pulcherimus				[2]	[1]
An. (Ano.) sacharovi	[herein]	[1]	[1]	[3,4,6]	[1]
An. (Cel.) superpictus	[herein]	[1]	[1]	[3,4,7]	[1]
Cq. (Coq.) buxtoni					[1]
Cq. (Coq.) richiardii	[herein]		[1]	[3,4]	[1]
Cs. (Cus.) alaskaensis		[1]			[1]
Cs. (Cus.) annulata	[herein]	[1]	[1]	[4]	[1]
Cs. (Cus.) fumipennis		[1]	[1]		[1]
Cs. (All.) longiareolata	[herein]	[1]	[1]	[2,3,4,5]	[1]
Cs. (Cus.) morsitans		[1]	[1]		[1]
Cs. (Cus.) subochrea	[herein]		[1]	[3,4]	[1]
Cx. (Mai.) deserticola					[1]
Cx. (Mai.) hortensis	[herein]	[1]	[1]	[2,4,5]	[1]
Cx. (Ncx.) impudicus					[1]
Cx. (Cux.) laticinctus			[1]		[1]
Cx.(Ncx.) martinii	[herein]				[1]
Cx. (Cux.) mimeticus	[herein]	[1]	[1]	[2,5]	[1]
Cx. (Bar.) modestus	[herein]	[1]	[1]	[2,4,5]	[1]
Cx. (Cux) perexiguus				[5]	[1]
Cx. (Cux.) pipiens	[herein]	[1]	[1]	[2,3,4,5]	[1]
Cx. (Bar.) pusillus					[1]
Cx. (Ncx.) territans	[herein]	[1]	[1]		[1]
Cx. (Cux.) theileri	[herein]	[1]	[1]	[2,3,4,5]	[1]
Cx. (Cux.) torrentium	[herein]			[4]	[1]
Cx. (Cux.) tritaeniorhynchus		[1]	[1]	[3,4,5]	[1]
Or. pulcripalpis			[1]		[1]
Ur. (Pfc.) unguiculata	[herein]	[1]	[1]	[2,3,4]	[1]
Total species	28	33	31	24	62

References for ~~the~~ Table S5

1. Robert V, Günay F, Le Goff G, Boussès P, Sulesco T, Khalin A, Medlock J, Kampen H, Petrić D, Schaffner F. 2019. Distribution chart for Euro-Mediterranean mosquitoes (western Palaearctic region). Journal of the European Mosquito Control Association, 37, 1-28.
2. Khoshdel-Nezamiha F, Vatandoost H, Azari-Hamidian S, Bavani MM, Dabiri F, Entezar-Mahdi R, Chavshin AR. 2014. Fauna and larval habitats of mosquitoes (Diptera: Culicidae) of West Azerbaijan Province, Northwestern Iran. Journal of Arthropod-Borne Diseases, 8(2), 163-173.

3. Abai MR, Azari-Hamidian S, Ladonni H, Hakimi M, Mashhadi-Esmail K, Sheikhzadeh K, Kousha A, Vatandoost H. 2007. Fauna and checklist of mosquitoes (Diptera: Culicidae) of East Azerbaijan Province, Northwestern Iran. Iranian Journal of Arthropod-Borne Diseases, 1(2), 27-33
4. Azari-Hamidian S, Yaghoobi-Ershadi MR, Javadian E, Abai MR, Mobedi I, Linton YM. 2009. Distribution and ecology of mosquitoes in a focus of dirofilariasis in northwestern Iran, with the first finding of filarial larvae in naturally infected local mosquitoes. Medical and Veterinary Entomology, 23, 111-121.
5. Moradi-Asl E, Hazrati S, Vatandoost H, Emdadi D, Ghorbani E, Ghasemian A, Rafiee M, Panahi A, Shokri A. 2018. Fauna and larval habitat characteristics of mosquitoes (Diptera: Culicidae) in Ardabil Province, northwestern Iran. Journal of Health, 9(3), 259-266. [in Iranian]
6. Oshagi MA, Vatandoost H, Gorouhi A, Abai MR, Madjidpour A, Arshi S, Sadeghi H, Nazari M, Mehravaran A. 2011. Anopheline species composition in borderline of Iran-Azerbaijan. Acta Tropica, 119, 44-49.
7. Oshagi MA, Shemshad Kh, Yaghobi-Ershadi MR, Pedram M, Vatandoost H, Abaie MR, Akbarzadeh K, Mohtarami F. 2007. Genetic structure of the malaria vector *Anopheles superpictus* in Iran using mitochondrial cytochrome (COI and COII) and morphologic markers: A new species complex? Acta Tropica, 101, 241-248.
